# Cytogenetic and pathologic characterization of *MYC*-rearranged B-cell lymphomas in pediatric and young adult patients

**DOI:** 10.1007/s12308-024-00579-6

**Published:** 2024-04-02

**Authors:** Marie-France Gagnon, Frido K. Bruehl, Daniel R. Sill, Reid G. Meyer, Patricia T. Greipp, Nicole L. Hoppman, Xinjie Xu, Linda B. Baughn, Jess F. Peterson, Ellen D. McPhail, Rhett P. Ketterling, Rebecca L. King

**Affiliations:** 1 Division of Laboratory Genetics and Genomics, Department of Laboratory Medicine and Pathology, Mayo Clinic, Rochester, MN USA; 2 Division of Hematopathology, Department of Laboratory Medicine and Pathology, Mayo Clinic, Rochester, MN USA

**Keywords:** Pediatric B-cell lymphoma, Young adult B-cell lymphoma, MYC rearrangement, B-cell lymphoma, FISH, Double-hit cytogenetics, Burkitt lymphoma, High-grade B-cell lymphoma

## Abstract

*MYC*-rearranged B-cell lymphoma (BCL) in the pediatric/young adult (YA) age group differs substantially in disease composition from adult cohorts. However, data regarding the partner genes, concurrent rearrangements, and ultimate diagnoses in these patients is scarce compared to that in adult cohorts. We aimed to characterize the spectrum of *MYC*-rearranged (*MYC*-R) mature, aggressive BCL in the pediatric/YA population. A retrospective study of morphologic, immunophenotypic, and fluorescence in situ hybridization (FISH) results of patients age ≤ 30 years with suspected Burkitt lymphoma (BL), diffuse large B-cell lymphoma (DLBCL) or high-grade B-cell lymphoma (HGBCL), and a *MYC*-R by FISH between 2013–2022 was performed. Two-hundred fifty-eight cases (129 (50%) pediatric (< 18 years) and 129 (50%) YA (18–30 years)) were included. Most *MYC*-R BCL in pediatric (89%) and YA (66%) cases were BL. While double-hit (DH) cytogenetics (*MYC* with *BCL2* and/or *BCL6*-R, HGBCL-DH) was rare in the pediatric population (2/129, 2%), HGBCL-DH increased with age and was identified in 17/129 (13%) of YA cases. Most HGBCL-DH had *MYC* and *BCL6*-R, while *BCL2*-R were rare in both groups (3/258, 1%). *MYC*-R without an IG partner was more common in the YA group (14/116 (12%) vs 2/128 (2%), *p* = 0.001). The pediatric to YA transition is characterized by decreasing frequency in BL and increasing genetic heterogeneity of *MYC*-R BCL, with emergence of DH-BCL with *MYC* and *BCL6*-R. FISH to evaluate for *BCL2* and *BCL6* rearrangements is likely not warranted in the pediatric population but should continue to be applied in YA BCL.

## Introduction

*MYC*-rearrangements occur in various B-cell lymphomas (BCL) including, amongst others, Burkitt lymphoma (BL), diffuse large B-cell lymphoma (DLBCL), and double-hit lymphoma (DHL), formally known as high-grade B-cell lymphoma (HGBCL) with *MYC* and *BCL2* rearrangements (HGBCL-DH-*BCL2*), and HGBCL with *MYC* and *BCL6* rearrangements (HGBCL-DH-*BCL6*) [1]. These entities have distinctive morphologic, immunophenotypic, and genetic features. BL is characterized by high-grade monomorphic cytology, a starry-sky growth pattern, CD10 and CD20 positivity, and *MYC* rearrangements, virtually always with an immunoglobulin (IG) partner. DHL is characterized by large cell or high-grade cytology with concurrent *MYC* rearrangements and *BCL2* and/or *BCL6* rearrangements. DLBCL, NOS exhibits large cell morphology with a mature B-cell phenotype and may also display isolated *MYC* rearrangements [1–3].

The frequency and distribution of aggressive BCL subtypes vary according to age. While DLBCL is the most common subtype identified in adult patients and DHL accounts for 8% of de novo DLBCL in this population [4], BL is the most frequently documented subtype in pediatric individuals, representing 30–50% of non-Hodgkin lymphoma (NHL) cases [3]. In contrast, DLBCL accounts for only 10–20% of NHL cases in the pediatric population [5, 6]. As such, the transition from the pediatric phase to the young adult (YA) phase, defined here as ages < 1–17 versus 18–30, respectively, is characterized by a shift in the spectrum of aggressive BCL, with a concurrent decrease in BL and increase in the frequency of other subtypes [6]. While BL may predominate among pediatric patients, the scope of additional *MYC*-rearranged mature aggressive BCLs remains poorly defined across the pediatric/YA spectrum.

In this study, we sought to provide a comprehensive morphologic, immunophenotypic, and genetic appraisal of *MYC*-rearranged (*MYC*-R) mature aggressive BCL in the pediatric/YA population.

## Materials and methods

### Study design and cohort

The study population included patients aged ≤ 30 years with suspected HGBCL, DLBCL, or BL with a *MYC*-R identified by fluorescence in situ hybridization (FISH) between 2013 and 2022 at Mayo Clinic, Rochester, MN. The cohort was divided into two groups based on age: the pediatric group included patients aged 1–17 years and the YA group comprised patients aged 18–30 years. Institutional Review Board approval was obtained for this study.

### Pathology

Available hematoxylin and eosin (H&E)-stained sections of diagnostic biopsy tissue were reviewed and classified according to morphologic and immunophenotypic criteria of the International Consensus Classification [1]. Large cell size was defined as tumor nuclei on average being larger than those of background histiocytes. Burkitt-like morphology was defined as having a monomorphic population of medium-sized cells with basophilic cytoplasm, round nuclei, and finely clumped nuclear chromatin, typically with a starry-sky background of tingible body macrophages. A designation of high-grade morphology was used for cases with intermediate-sized cells, typically with fine chromatin, but without definitive features of BL either because of increased nuclear pleomorphism, lack of tingible body macrophages in a well-sampled biopsy, or for morphology resembling lymphoblastic lymphoma (blastoid morphology). Immunophenotypically, a BL-like phenotype was defined as cases expressing CD10, CD20, and BCL6 without or with only very dim/subset expression of BCL2.

### Fluorescence in situ hybridization analysis

FISH analysis was performed as part of diagnostic evaluation using laboratory-defined algorithms or based on a pathologist’s request of specific probes. *MYC* FISH was performed using concurrent *MYC* break-apart (BAP) and IGH/*MYC* dual color, dual fusion (DF) probe strategies (Abbott Laboratories, Des Plaines, IL) in the setting of DLBCL, HGBCL, or BL. IGK/*MYC* and IGL/*MYC* DF probe sets were performed using laboratory developed tests. These were performed either at pathologist discretion up front, or as a reflex in the setting of a *MYC*-R without an IGH partner per laboratory protocol. Cases with a positive *MYC*-R with any probe strategy were considered as *MYC*-R and underwent testing with *BCL2* and *BCL6* BAP probes (Abbott Laboratories) if these were not already performed up front. Where material was available, additional FISH probes were performed retrospectively for these probe sets if not performed at diagnosis.

All FISH analysis was performed on formalin-fixed, paraffin-embedded (FFPE) or fresh tissue using standard FISH pretreatment, hybridization, and fluorescence microscopy in accordance with specimen-specific laboratory protocols [7]. FFPE samples were conserved at room temperature. Fresh blood/bone marrow and tissue were respectively kept in anticoagulant and balanced salt solution at ambient or refrigerated temperature. A minimum of one hundred interphase nuclei for BAP probe strategies and two hundred nuclei for DF strategies were scored using previously described cutoffs [8, 9]. FISH analysis was performed by at least two qualified clinical cytogenetic technologists and interpreted by a board-certified (American Board of Medical Genetics and Genomics) clinical cytogeneticist.

### Statistical methods

Categorical variables are presented as frequencies and percentages and continuous variables as means and standard deviations or medians and ranges. Chi-square tests or Fisher exact probability tests were used to assess between-group differences in categorical variables and non-parametric tests such as Kruskal–Wallis *H* and Wicoxon rank sum test were used for non-Gaussian variables. Logistic regression analyses were used to assess the frequency of DH-cytogenetics adjusting for age. All statistical tests were two-sided with a significance level of 0.05. Analyses were performed with R [10] version 4.2.1 using the dplyr [11], ggplot2 [12], and gtsummary [13] packages.

## Results

### Study cohort

The study cohort comprised 258 pediatric/YA cases, including 129 (50%) pediatric patients and 129 (50%) YA patients (Table 1). Median age in the study population was 17.5 years (range 1–30) and 194 (75%) were male. When considering patients from the pediatric group, median age was 9 years (interquartile range (IQR) 5–13) and 105 (81%) were male. Among YA patients, median age was 25 (IQR 21–28) and 89 (69%) were male.

**Table 1:** Cytogenetic, immunophenotypic, and B-cell lymphoma subtype according to age group

Characteristic	All cohort, *N* = 258	Pediatric, *N* = 129^1^	Young adult, *N* = 129^1^	*p* value^2^
Demographics (258 evaluable)				
Age	17.5 (9,25)	9 (5, 13)	25 (21, 28)	< 0.001
Male gender	194/258 (75%)	105/129 (81%)	89/129 (69%)	0.021
MYC rearrangement partner (244 evaluable)				
All IG partner	228/244 (93%)	126/128 (98%)	102/116 (88%)	0.001
IGH	201/244 (82%)	112/128 (88%)	89/116 (77%)	0.03
IGL	20/244 (8%)	11/128 (9%)	9/116 (8%)	0.8
IGK	7/244 (3%)	3/128 (2%)	4/116 (3%)	0.6
Non-IG partner	16/244 (6%)	2/128 (2%)	14/116 (12%)	0.001
Concurrent rearrangements (258 evaluable)				
* MYC* and *BCL2* rearrangements	3/258 (1%)	1/129 (1%)	2/129 (2%)	> 0.9
* MYC* and *BCL6* rearrangements	16/258 (6%)	1/129 (1%)	15/129 (12%)	< 0.001
DH cytogenetics	19/258 (7%)	2/129 (2%)	17/129 (13%)	< 0.001
Immunophenotypic features				
BCL2 expression (72 evaluable)	13/72 (18%)	1/34 (3%)	12/38 (32%)	0.002
BCL6 expression (68 evaluable)	62/68 (91%)	28/32 (88%)	34/36 (94%)	0.4
MYC expression (41 evaluable)	39/41 (95%)	17/17 (100%)	22/24 (92%)	0.5
Diagnosis (138 evaluable)				
BL	107/138 (78%)	62/70 (89%)	45/68 (66%)	0.002
DHL	9/138 (7%)	0/70 (0%)	9/68 (13%)	0.001
DLBCL	20/138 (14%)	7/70 (10%)	13/68 (19%)	0.1
Other	2/138 (1%)	1/70 (1.4%)	1/68 (1.5%)	1

Pediatric < 1–17 years of age, young adult 18–30 years of age. *p* values refer to the comparison of the pediatric and young adult groups

*DH* double hit, *BL* Burkitt lymphoma, *DHL* double-hit lymphoma, *DLBCL* diffuse large B-cell lymphoma, *IG* immunoglobulin

^1^Median (IQR);* n* (%)

^2^Wilcoxon rank sum test; Pearson’s Chi-squared test; Fisher’s exact test

### FISH analysis results

A *MYC*-R was detected in 242/256 (94.5%) cases subjected to the *MYC* BAP (the *MYC* BAP probe was not performed in 2 cases). In the remaining 14/256 cases, the *MYC* BAP probe rendered equivocal results in 7 (3%) cases and normal results in 7 (3%) yet exhibited an IGH/*MYC* fusion by DF FISH. This indicates a 3% false negative (FN) rate of the *MYC* BAP probe in our cohort and a 5.5% FN rate if equivocals are considered negative. Among the 244 (95%) cases with complete assessment of IG rearrangement partners, an IGH partner was identified in 201 cases (82%). IGK and IGL partners were identified in 7 (3%) cases and 20 (8%) cases, respectively. No IG partner was identified in 16 (6%) cases. The remaining 14 cases did not have complete assessment of IG partners by DF FISH, precluding definitive characterization of *MYC*-R partners (*MYC*/IGK DF probe set not performed in 1 case, *MYC*/IGL DF probe set not performed in 1 case, both *MYC*/IGK and *MYC*/IGL DF probe sets not performed in 12 cases). In addition to the MYC-R, a rearrangement of *BCL2* was identified in 3/258 cases (1%) while 16 (6%) cases harbored a rearrangement of *BCL6*. No case displayed triple-hit cytogenetics (concurrent *MYC*, *BCL2*, and *BCL6* rearrangements) in our cohort.

**Fig. 1:**
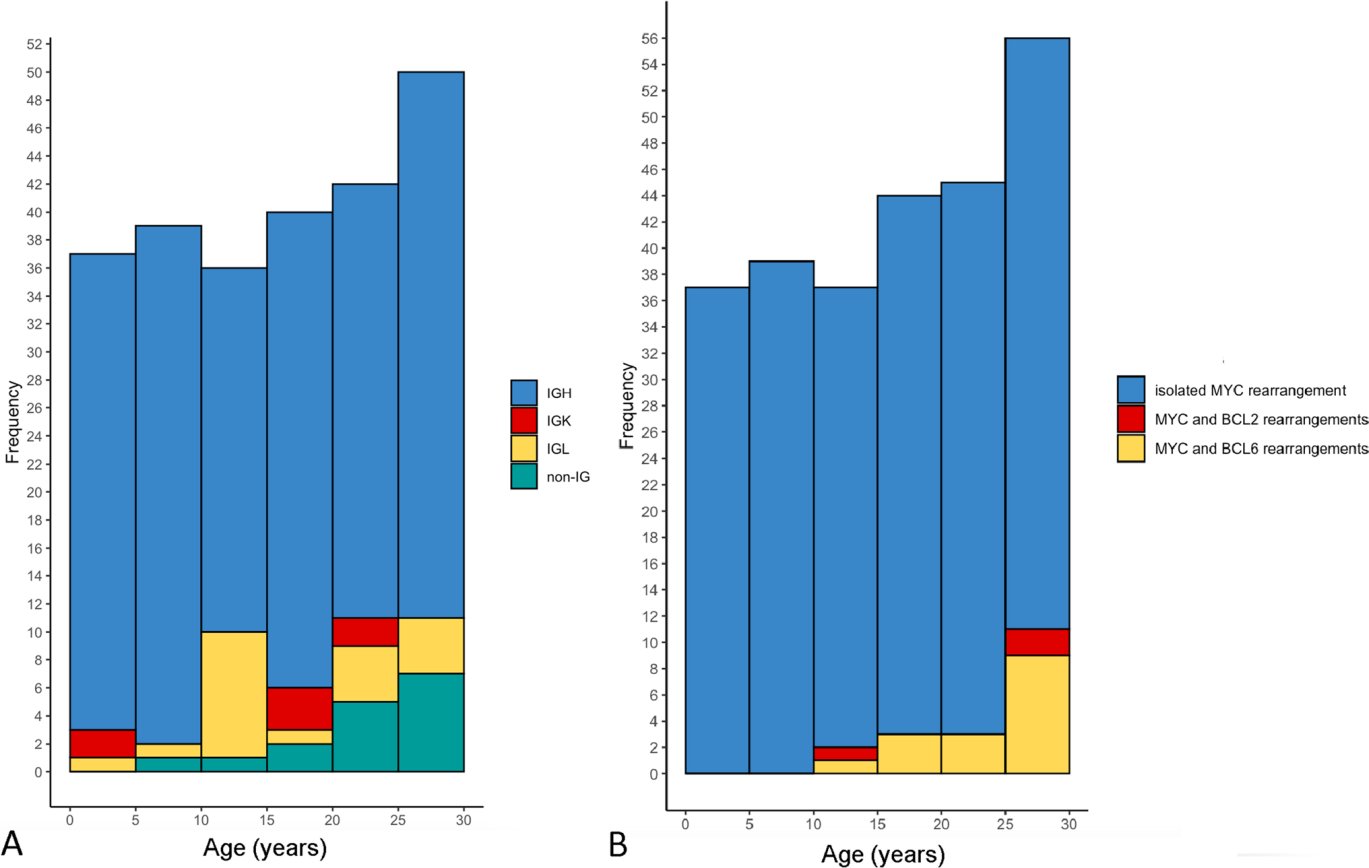
Cytogenetic features of study cohort according to age. **A** Distribution of *MYC* rearrangement partner according to age. **B** Frequency of *BCL2* and *BCL6* rearrangements in our cohort according to age

The distribution of *MYC*-R partners as identified by FISH was then compared between pediatric and YA patients in whom all three IG probe sets were performed (128 pediatric patients and 116 YA patients). An increased frequency of IG partners was found in the pediatric group in comparison with the YA group (126/128, 98% vs 102/116, 88% respectively, *p* = 0.001). Specifically, IGH rearrangements were more common in the pediatric group (112/128 (88%) vs 89/116 (77%), *p* = 0.03)). IGK frequency did not differ between the two groups (3/128 (2%) and 4/116 (3%) respectively, *p* = 0.6), nor did IGL frequency (11/128 (9%) vs 9/116 (8%), *p* = 0.8). The absence of an IG partner occurred less often in the pediatric group compared with the YA group (2/128 (2%) and 14/116 (12%), *p* = 0.001) in keeping with the higher rate of IGH partners in the pediatric group (Fig. 1A).

The frequency of HGBCL-DH-*BCL2* and HGBCL-DH-*BCL6* was also assessed in the pediatric and YA groups. HGBCL-DH-*BCL2* was rare in both pediatric and YA patients (1/129 (1%) and 2/129 (2%), respectively, *p* > 0.9). HGBCL-DH-*BCL2* occurred in a single pediatric patient (11-year-old male) and in two YA patients (27-year-old male and 30-year-old female). The concurrent *MYC*-R in cases with a *BCL2* rearrangement involved an IGL partner in the pediatric case and an IGH and non-IG partner in the YA cases. HGBCL-DH-*BCL6* was rare in the pediatric group (1/129, 1%) versus the YA group (15/129, 12%) (*p* < 0.001). HGBCL-DH-*BCL6* was identified in a 14-year-old individual in the pediatric group and in individuals aged 19 to 29 in the YA group. The *MYC*-R in cases with a *BCL6* rearrangement involved an IGL partner in the pediatric case and IGH (5/15), IGL (1/15) and non-IG partner (5/15) in the YA cases (IGK/MYC and IGL/MYC DF probe sets not performed in 4/15 YA cases). In total, the frequency of DH cytogenetics was significantly lower in pediatric vs YA cases (respectively 2/129 (2%) and 17/129 (13%), *p* < 0.001). Furthermore, the frequency of DH cytogenetics increased statistically significantly with increasing age (coefficient estimate 0.14, *p* < 0.001) (Fig. 1B).

### Morphologic and genetic correlation

**Fig. 2:**
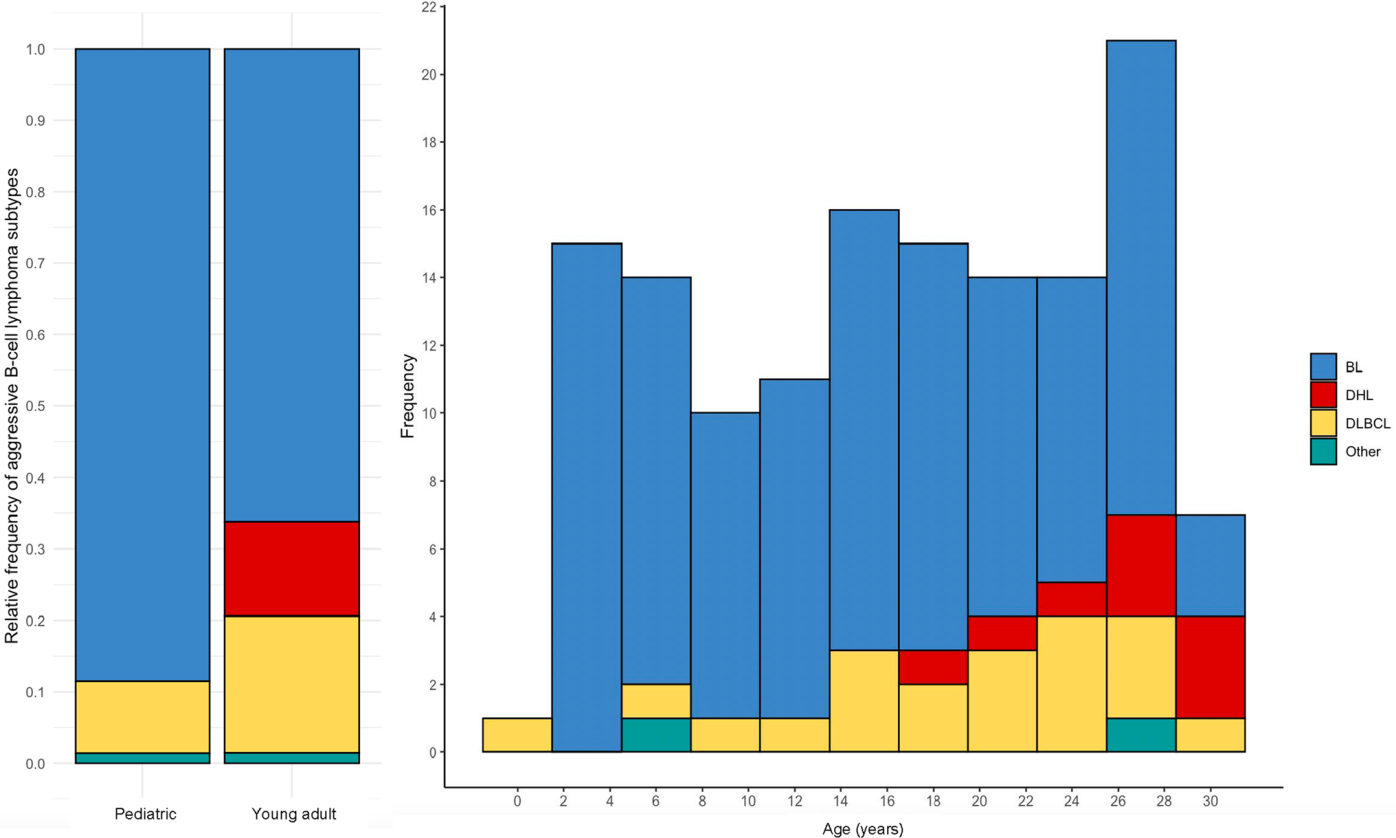
Distribution of B-cell lymphoma subtypes of study cohort according to age

**Fig. 3:**
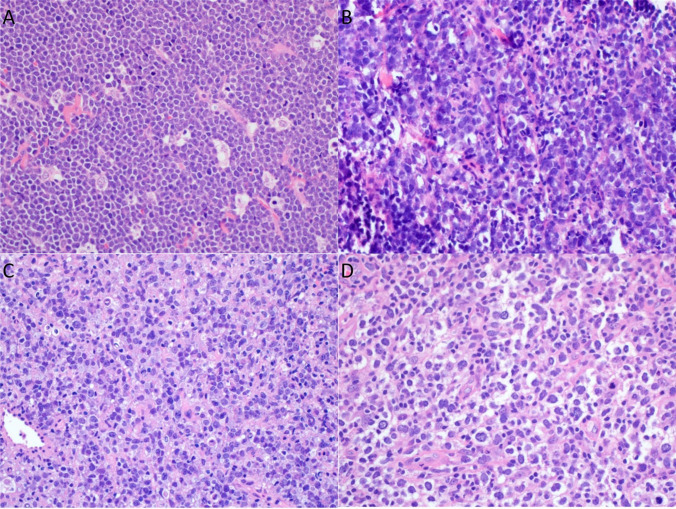
Examples of morphologic and genetic correlation from the study cohort (all H&E, 400 ×). **A** Burkitt lymphoma.case with Burkitt morphology and phenotype and *MYC*::IGH. **B** High-grade B-cell lymphoma with *MYC* and *BCL2* rearrangements. Case with non-Burkitt high-grade morphology, non-Burkitt phenotype, *MYC*::IGH and *BCL2* rearrangement. **C** High-grade B-cell lymphoma with *MYC* and *BCL6* rearrangements. Case with non-Burkitt high-grade morphology, Burkitt-phenotype, *MYC* and *BCL6* rearrangement. **D** Diffuse large B-cell lymphoma. Case with large cell morphology, Burkitt phenotype, *MYC* rearrangement with non-IG partner

In our study cohort, 138 cases had slides available for retrospective pathology review. Taking into account morphologic, phenotypic and genetic features, the following diagnoses were rendered: BL (*N* = 107; 78%), DLBCL (*N* = 20; 14%), and HGBCL with *MYC* and *BCL2* rearrangements or HGBCL with *MYC* and *BCL6* rearrangements (*N* = 9; 7%) (Table 2, Fig. 2). Examples of morphologic and genetic correlation and final diagnoses are illustrated in Fig. 3. While BL was the most common diagnosis in both pediatric (89%) and YA (66%) age groups, a significantly higher proportion of pediatric *MYC-*R B-cell lymphomas were BL (*p* = 0.002). Most BL cases (49/52; 94%) had a typical phenotype (CD10 + , *BCL6* + , *BCL2-*). The rearrangement partner was usually IGH (94/104, 90%), while IGL (7/104, 7%), IGK (2/104, 2%) and no IG partners (1/104, 1%) were found less frequently. Most DLBCL cases had a germinal center B-cell phenotype per the Hans algorithm (12/13, 92%). In 7/20 (35%) DLBCLs, an immunophenotypic profile compatible with BL was present, yet morphologic attributes precluded a diagnosis of BL (3 pediatric and 4 YA patients). Of these 7 DLBCL with a BL immunophenotype, 4 had *MYC*::IGH, one IGL::*MYC*, one had a non-IG partner, and one case was incompletely assessed (no IGL and IGK BAP probes). *MYC* rearrangement partners among cases with a diagnosis of DLBCL and complete assessment of IG partners by FISH were IGH (8/16; 50%), IGL (2/16; 13%), and IGK (1/16, 6%). No IG partner was detected in 5/16 (31%). The mediastinum was the biopsy site in 3/20 (15%) DLBCL, and all three occurred in YA. These may represent primary mediastinal large B-cell lymphoma, but due to lack of further clinical, imaging, or gene expression profiling information this remains speculative. One of these three had an IGL partner, and the *MYC* partner was not determined in two cases.

**Table 2:** Cytogenetic, cytomorphologic, and immunophenotypic features according to B-cell lymphoma subtype

Characteristic	BL, *N* = 107^1^	DHL, *N* = 9^1^	DLBCL, *N* = 20^1^	Other, *N* = 2^1^	*p* value^2^
Demographics					
Age group					< 0.001
Pediatric	62/107 (58%)	0/9 (0%)	7/20 (35%)	1/2 (50%)	
Young adult	45/107 (42%)	9/9 (100%)	13/20 (65%)	1/2 (50%)	
Mean age	15 (8, 22)	26 (25, 29)	20 (15, 24)	16 (11, 22)	0.001
Male gender	82/107 (77%)	3/9 (33%)	10/20 (50%)	2/2 (100%)	0.004
*MYC* rearrangement partner					< 0.001
All IG partner	103/104 (99%)	4/4 (100%)	11/16 (68%)	2/2 (100%)	
IGH	94/104 (90%)	3/4 (75%)	8/16 (50%)	2/2 (100%)	
IGL	7/104 (7%)	1/4 (25%)	2/16 (13%)	0/2 (0%)	
IGK	2/104 (2%)	0/4 (0%)	1/16 (6%)	0/2 (0%)	
No IG partner	1/104 (1.0%)	0/4 (0%)	5/16 (31%)	0/2 (0%)	
Concurrent rearrangements					
* BCL2* rearrangements	0/107 (0%)	2/9 (22%)	0/20 (0%)	0/2 (0%)	0.006
* BCL6* rearrangements	0/107 (0%)	7/9 (78%)	0/20 (0%)	0/2 (0%)	< 0.001
Cytomorphologic and immunophenotypic features					
Starry sky pattern	91/107 (85%)	2/9 (22%)	3/20 (15%)	0/2 (0%)	< 0.001
CD20 expression	70/70 (100%)	6/6 (100%)	15/15 (100%)	2/2 (100%)	Not applicable
CD10 expression	64/64 (100%)	4/6 (67%)	12/14 (86%)	2/2 (100%)	0.004
BCL6 expression	48/51 (94%)	4/6 (67%)	10/11 (91%)	0/0 (NA%)	0.10
MUM1 expression	7/31 (23%)	3/4 (75%)	5/11 (45%)	0/0 (NA%)	0.057
BCL2 expression	3/53 (6%)	5/7 (71%)	4/11 (36%)	1/1 (100%)	< 0.001
MYC expression	32/32 (100%)	3/4 (75%)	3/4 (75%)	1/1 (100%)	0.044
TdT expression	0/28 (0%)	0/1 (0%)	0/4 (0%)	1/1 (100%)	0.059
EBER positivity	6/19 (32%)	0/4 (0%)	1/6 (17%)	0/0 (NA%)	0.6
Ki67	100 (95, 100)	90 (85, 93)	85 (70, 95)	97 (96, 98)	< 0.001

*BL* Burkitt lymphoma, *DHL* double-hit lymphoma, *DLBCL* diffuse large B-cell lymphoma, *IG* immunoglublin

^1^*n* (%); median (IQR)

^2^Fisher’s exact test; Kruskal–Wallis rank sum test

Nine cases had DHL cytogenetics, all of which occurred in YA patients (2 HGBCL-DH-*BCL2 and 7* HGBCL-DH-*BCL6*). Of the HGBCL-DH-*BCL2*, one had high grade and one had large cell morphology and the one with evaluable stains was GCB phenotype. Of the HGBCL-DH-*BCL6,* one had high grade (14%) and 6 (86%) had large cell morphology; Hans algorithm on these 7 showed 3 GCB, 2 non-GC, and 2 not available. A BL-like immunophenotype was observed in 1/7 (14%) HGBCL-DH-*BCL6* with available information. Of the 4 DHL cases with complete assessment for IG rearrangement partners, 3 (75%) had an IGH partner and 1 (25%) had an IGL partner. No IGK partner was observed.

EBV in situ hybridization (EBER) was performed on 29/138 (21%) cases with available biopsy tissue slides for pathologic review. EBER was positive in 6/19 (32%) cases of BL, 0/4 (0%) of HGBCL and 1/6 (17%) of DLBCL. Among EBV-positive BL, four occurred in the pediatric, and two in the YA population, and all showed IGH partners.

Pathologic review of the biopsy material revealed two unusual cases of *MYC*-R B-cell lymphoma: a case of a 6-year-old boy with BL-like morphology, isolated *MYC:*:IGH rearrangement, CD10 positivity and a high Ki67 proliferative index (> 95%) showed TdT positivity. Cases like this are now best considered B lymphoblastic leukemia/lymphoma based on recent genetic studies [1, 14]. Another case (27-year-old male) with IGH::*MYC* rearrangement showed HGBCL morphology without Burkitt-like features and with expression of CD10 and strong BCL2, but without *BCL2* or *BCL6* rearrangements; it was felt this was best categorized as HGBCL, NOS.

## Discussion

As an endeavor to address the information gap regarding the scope and distribution of *MYC*-R mature aggressive BCL subtypes in the pediatric and YA population, we provide a pathologic and genetic assessment *MYC*-R, mature aggressive BCL in a large cohort. We illustrate that while the majority of *MYC*-R BCL in pediatric and YA patients are BL, and that DH cytogenetics are rare in the pediatric group, the frequency of DHL rises with increasing age and DH cytogenetics in YA are predominantly HGBCL-DH-*BCL6*. Furthermore, the false negative rate of MYC BAP alone in our cohort was up to 5.5%, in line with previously published data in adult cohorts and further supporting the use of both *MYC* BAP and *MYC*::IGH probes in routine practice in all age groups [8, 9].

Mature aggressive BCL with a *MYC*-R encompass a biologically and clinicopathologically heterogeneous group of disorders. In line with epidemiology considerations and concerns related to chemotherapy toxicity, therapeutic management approaches vary between pediatric and YA patients. Distinctly, in adults with DLBCL/HGBCL, anthracycline-based regimens with cytogenetic risk-adapted therapy are standard practice [15]. As DH cytogenetics portend poorer response and overall-survival rates with R-CHOP therapy [16, 17], higher intensity regimens such as a DA-EPOCH-R are routinely used in adult patients in this setting [15]. In contrast, pediatric patients with aggressive BCL are generally treated with chemotherapy regimens designed for BL regardless of morphology including Berlin-Frankfurt-Münster (BFM) and LMB-96 based regimens [6]. While excellent outcomes have been reported with BL-regimens in this population [6], a scarcity of data has nonetheless precluded a refined assessment of the significance of DH cytogenetics and preferred therapeutic options across the pediatric to YA transition [18].

The relationship between age and genomic attributes in mature aggressive BCL is complex and not fully elucidated. Current evidence suggests that the biology of BL in YA patients may not differ significantly from that of pediatric patients and these two groups have been found to share significant molecular homogeneity [19, 20]. In contrast, the biology of DLBCL may be more influenced by age. Diverging pathogenetic mechanisms of disease have been supported by distinct genetic alterations such as the recurrent loss of 4p16 and 19q13.32 and gain of 16p11.2 in pediatric DLCBL compared with adult DLBCL [21]. Further, the genomic complexity of DLBCL appears to augment with increasing age. In fact, a gradual increment in the genomic complexity of DLBCL observed with age was described as consistent with an “age evolution model” by Klapper et al. [22]. While different in scope, our results remain in line with this model as a significant increase in the heterogeneity of *MYC* rearrangement partners and in the frequency of DH cytogenetics with age is documented.

The genetic hallmark of BL is a translocation juxtaposing *MYC* with an IG locus, most often IGH (80%), but also IGL and IGK [23]. In contrast to BL, DLBCL/HGBCL exhibits more heterogeneity with regards to *MYC* rearrangement partners. While IG partners are documented in 48–63% of cases, no IG partner is found in 37–52% [24–26]. Several non-IG partners have been described including *PAX5, BCL6, BCL11A, IKZF1*, and *SOCS1* [27, 28]. In line with these considerations, *MYC* was rearranged with an IG partner in nearly all BL cases in our cohort (99%), whereas 31% of DLBCL cases had a non-IG partner. When considering the spectrum of *MYC*-R partners according to age groups, a statistically significant increase in cases without an IG partner was identified among YA cases (12% vs 2% in the pediatric group, *p* = 0.001). The prognostic implications of *MYC*-R with non-IG partners remain contentious, and while the prognostic significance of non-IG partners has been called into question [16, 24], the increased frequency of cases with no IG partner in YA patients and in DLBCL cases remains in keeping with increased heterogeneity of disease with advancing age across the pediatric/YA spectrum.

In our study cohort, DH cytogenetics were rarely documented in the pediatric population (2%). These results mirror those identified in a cohort of 50 pediatric patients with DLBCL derived from the German Berlin-Frankfurt-Munster multicenter trial by Oschlies et al. in which no *BCL2* rearrangements were identified [29]. In contrast, DHL was identified in 13% of YA patients in our study. YA DHL is predominantly HGBCL-DH-*BCL6* (15/17 DHL cases, 88%), contrasting the figures documented in adult populations in whom HGBCL-DH-BCL2 prevail, accounting for 80–90% of DH/TH lymphomas [30].

While the 5th edition WHO has eliminated HGBCL with *MYC* and *BCL6* rearrangements as a diagnostic entity [2], the International Consensus Classification separates HGBCL-DH-*BCL2* yet retains HGBCL-DH-*BCL6* as a provisional entity [1]. While there is clear biologic data to support separating these two entities [4, 31], the clinical significance and biology of HGBCL-DH-*BCL6* remains understudied. Data is limited, but some studies do suggest benefit from more aggressive therapy regimens in patients with HGBCL-DH-*BCL6,* arguing that more data is needed before elimination of this category [24, 26, 32–35]. Additionally, in up to 30% of cases of HGBCL-DH-*BCL6*, *MYC*-R involve the *BCL6* gene as the rearrangement partner [30, 36]. This profile has been described as “pseudo-double hit” [37]. Our study design did not allow for specific identification of this phenomenon. While HGBCL-DH-*BCL6* lymphomas appear molecularly and prognostically heterogeneous [24, 26, 30, 33–36, 38, 39], potential distinct clinico-biologic correlates of “pseudo-double hit” BCL remain poorly studied [36, 40, 41]. Accordingly, further data is needed to assess the clinical relevance of genetic testing to identify *BCL6::MYC* rearrangements.

We speculate that distinguishing HGBCL-DH-*BCL6* from DLBCL, NOS may prove even more relevant in YA patients who are more likely to be eligible for and tolerate intensive chemotherapy regimens than older adults. Our findings support continuing to evaluate for HGBCL-DH-BCL6 in the YA cohort, although further data is needed to determine the best therapeutic options for these patients.

Given the rarity of their occurrence, whether to perform *BCL2* and *BCL6* FISH probes in the pediatric age group remains an open question, and one which our study alone cannot fully answer. Our data suggest that minimal harm would be done if these were not performed, especially since pediatric patients with mature aggressive BCL are generally treated with BL-like regimens regardless of subtype [6].

Our study has limitations related to the use of a reference laboratory cohort with somewhat limited access to tissue specimens and absence of outcome data. Since cases in our cohort were identified based on pathologist-initiated FISH testing and our study focuses specifically on those with *MYC*-R, we are unable to discuss the incidence of *MYC* rearrangement in the P/YA population.

In conclusion, our study provides a large-scale assessment of the spectrum of *MYC*-R BCL in pediatric and YA patients and of FISH analysis results for *MYC, BCL2, BCL6* rearrangements and *MYC* IG rearrangement partners. These data directly inform clinical practice with regard to FISH testing and probe selection, and lay groundwork for further studies looking at the clinical significance of *MYC* partners and high-grade B-cell lymphoma in the pediatric and YA population.

## References

[1] Campo E, Jaffe ES, Cook JR, Quintanilla-Martinez L, Swerdlow SH, Anderson KC, Brousset P, Cerroni L, de Leval L, Dirnhofer S, Dogan A, Feldman AL, Fend F, Friedberg JW, Gaulard P, Ghia P, Horwitz SM, King RL, Salles G, San-Miguel J, Seymour JF, Treon SP, Vose JM, Zucca E, Advani R, Ansell S, Au WY, Barrionuevo C, Bergsagel L, Chan WC, Cohen JI, d'Amore F, Davies A, Falini B, Ghobrial IM, Goodlad JR, Gribben JG, Hsi ED, Kahl BS, Kim WS, Kumar S, LaCasce AS, Laurent C, Lenz G, Leonard JP, Link MP, Lopez-Guillermo A, Mateos MV, Macintyre E, Melnick AM, Morschhauser F, Nakamura S, Narbaitz M, Pavlovsky A, Pileri SA, Piris M, Pro B, Rajkumar V, Rosen ST, Sander B, Sehn L, Shipp MA, Smith SM, Staudt LM, Thieblemont C, Tousseyn T, Wilson WH, Yoshino T, Zinzani PL, Dreyling M, Scott DW, Winter JN, Zelenetz AD (2022). The International Consensus Classification of Mature Lymphoid Neoplasms: a report from the Clinical Advisory Committee. Blood.

[2] Alaggio R, Amador C, Anagnostopoulos I, Attygalle AD, Araujo IBO, Berti E, Bhagat G, Borges AM, Boyer D, Calaminici M, Chadburn A, Chan JKC, Cheuk W, Chng WJ, Choi JK, Chuang SS, Coupland SE, Czader M, Dave SS, de Jong D, Du MQ, Elenitoba-Johnson KS, Ferry J, Geyer J, Gratzinger D, Guitart J, Gujral S, Harris M, Harrison CJ, Hartmann S, Hochhaus A, Jansen PM, Karube K, Kempf W, Khoury J, Kimura H, Klapper W, Kovach AE, Kumar S, Lazar AJ, Lazzi S, Leoncini L, Leung N, Leventaki V, Li XQ, Lim MS, Liu WP, Louissaint A, Jr., Marcogliese A, Medeiros LJ, Michal M, Miranda RN, Mitteldorf C, Montes-Moreno S, Morice W, Nardi V, Naresh KN, Natkunam Y, Ng SB, Oschlies I, Ott G, Parrens M, Pulitzer M, Rajkumar SV, Rawstron AC, Rech K, Rosenwald A, Said J, Sarkozy C, Sayed S, Saygin C, Schuh A, Sewell W, Siebert R, Sohani AR, Tooze R, Traverse-Glehen A, Vega F, Vergier B, Wechalekar AD, Wood B, Xerri L, Xiao W (2022) The 5th edition of the World Health Organization Classification of Haematolymphoid Tumours Lymphoid Neoplasms. Leukemia 36:1720–1748 10.1038/s41375-022-01620-2 10.1038/s41375-022-01620-2 PMC9214472 35732829

[3] Swerdlow SH, Campo E, Harris NL, Jaffe ES, Pileri SA, Stein H, Thiele J (2017) WHO classification of tumours of haematopoietic and Lymphoid Tissues. Revised 4th ed. Lyon: IARC

[4] Scott DW, King RL, Staiger AM, Ben-Neriah S, Jiang A, Horn H, Mottok A, Farinha P, Slack GW, Ennishi D, Schmitz N, Pfreundschuh M, Nowakowski GS, Kahl BS, Connors JM, Gascoyne RD, Ott G, Macon WR, Rosenwald A (2018). High-grade B-cell lymphoma with MYC and BCL2 and/or BCL6 rearrangements with diffuse large B-cell lymphoma morphology. Blood.

[5] Miles RR, Raphael M, McCarthy K, Wotherspoon A, Lones MA, Terrier-Lacombe MJ, Patte C, Gerrard M, Auperin A, Sposto R, Davenport V, Cairo MS, Perkins SL, Group SLCUNS (2008). Pediatric diffuse large B-cell lymphoma demonstrates a high proliferation index, frequent c-Myc protein expression, and a high incidence of germinal center subtype: Report of the French-American-British (FAB) international study group. Pediatr Blood Cancer.

[6] Sandlund JT, Martin MG (2016). Non-Hodgkin lymphoma across the pediatric and adolescent and young adult age spectrum. Hematology Am Soc Hematol Educ Program.

[7] Mascarello JT, Hirsch B, Kearney HM, Ketterling RP, Olson SB, Quigley DI, Rao KW, Tepperberg JH, Tsuchiya KD, Wiktor AE, Working Group of the American College of Medical Genetics Laboratory Quality Assurance C (2011). Section E9 of the American College of Medical Genetics technical standards and guidelines: fluorescence in situ hybridization. Genet Med.

[8] King RL, McPhail ED, Meyer RG, Vasmatzis G, Pearce K, Smadbeck JB, Ketterling RP, Smoley SA, Greipp PT, Hoppman NL, Peterson JF, Baughn LB (2019). False-negative rates for MYC fluorescence in situ hybridization probes in B-cell neoplasms. Haematologica.

[9] Peterson JF, Pitel BA, Smoley SA, Vasmatzis G, Smadbeck JB, Greipp PT, Ketterling RP, Macon WR, Baughn LB (2019) Elucidating a false-negative MYC break-apart fluorescence in situ hybridization probe study by next-generation sequencing in a patient with high-grade B-cell lymphoma with IGH/MYC and IGH/BCL2 rearrangements. Cold Spring Harb Mol Case Stud 5. 10.1101/mcs.a004077 10.1101/mcs.a004077 PMC6549546 31160360

[10] R Core Team (2023) R: a language and environment for statistical computing. R foundation for statistical computing, Vienna, Austria. https://www.R-project.org

[11] Wickham H, François R, Henry L, Müller K, Vaughan D (2023) dplyr: A grammar of data manipulation. R package version 1.1.4. https://github.com/tidyverse/dplyr

[12] Wickham H (2016). ggplot2: Elegant Graphics for Data Analysis.

[13] Daniel D, Sjoberg KW, Curry M, Lavery JA, Larmarange J (2021). Reproducible summary tables with the gtsummary package. The R Journal.

[14] Wagener R, Lopez C, Kleinheinz K, Bausinger J, Aukema SM, Nagel I, Toprak UH, Seufert J, Altmuller J, Thiele H, Schneider C, Kolarova J, Park J, Hubschmann D, Murga Penas EM, Drexler HG, Attarbaschi A, Hovland R, Kjeldsen E, Kneba M, Kontny U, de Leval L, Nurnberg P, Oschlies I, Oscier D, Schlegelberger B, Stilgenbauer S, Wossmann W, Schlesner M, Burkhardt B, Klapper W, Jaffe ES, Kuppers R, Siebert R (2018). IG-MYC (+) neoplasms with precursor B-cell phenotype are molecularly distinct from Burkitt lymphomas. Blood.

[15] Sehn LH, Salles G (2021). Diffuse large B-cell lymphoma. Reply N Engl J Med.

[16] Horn H, Ziepert M, Becher C, Barth TF, Bernd HW, Feller AC, Klapper W, Hummel M, Stein H, Hansmann ML, Schmelter C, Moller P, Cogliatti S, Pfreundschuh M, Schmitz N, Trumper L, Siebert R, Loeffler M, Rosenwald A, Ott G, German High-Grade Non-Hodgkin Lymphoma Study G (2013). MYC status in concert with BCL2 and BCL6 expression predicts outcome in diffuse large B-cell lymphoma. Blood.

[17] Savage KJ, Johnson NA, Ben-Neriah S, Connors JM, Sehn LH, Farinha P, Horsman DE, Gascoyne RD (2009). MYC gene rearrangements are associated with a poor prognosis in diffuse large B-cell lymphoma patients treated with R-CHOP chemotherapy. Blood.

[18] Jeon W, Koh YK, Kang S, Kim H, Koh KN, Im HJ (2022). Clinical characteristics and treatment outcomes of children and adolescents with aggressive mature B-cell lymphoma: a single-center analysis. Blood Res.

[19] Klapper W, Szczepanowski M, Burkhardt B, Berger H, Rosolowski M, Bentink S, Schwaenen C, Wessendorf S, Spang R, Moller P, Hansmann ML, Bernd HW, Ott G, Hummel M, Stein H, Loeffler M, Trumper L, Zimmermann M, Reiter A, Siebert R, Molecular Mechanisms in Malignant Lymphomas Network Project of the Deutsche K (2008). Molecular profiling of pediatric mature B-cell lymphoma treated in population-based prospective clinical trials. Blood.

[20] Thomas N, Dreval K, Gerhard DS, Hilton LK, Abramson JS, Ambinder RF, Barta S, Bartlett NL, Bethony J, Bhatia K, Bowen J, Bryan AC, Cesarman E, Casper C, Chadburn A, Cruz M, Dittmer DP, Dyer MA, Farinha P, Gastier-Foster JM, Gerrie AS, Grande BM, Greiner T, Griner NB, Gross TG, Harris NL, Irvin JD, Jaffe ES, Henry D, Huppi R, Leal FE, Lee MS, Martin JP, Martin MR, Mbulaiteye SM, Mitsuyasu R, Morris V, Mullighan CG, Mungall AJ, Mungall K, Mutyaba I, Nokta M, Namirembe C, Noy A, Ogwang MD, Omoding A, Orem J, Ott G, Petrello H, Pittaluga S, Phelan JD, Ramos JC, Ratner L, Reynolds SJ, Rubinstein PG, Sissolak G, Slack G, Soudi S, Swerdlow SH, Traverse-Glehen A, Wilson WH, Wong J, Yarchoan R, ZenKlusen JC, Marra MA, Staudt LM, Scott DW, Morin RD (2023). Genetic subgroups inform on pathobiology in adult and pediatric Burkitt lymphoma. Blood.

[21] Deffenbacher KE, Iqbal J, Sanger W, Shen Y, Lachel C, Liu Z, Liu Y, Lim MS, Perkins SL, Fu K, Smith L, Lynch J, Staudt LM, Rimsza LM, Jaffe E, Rosenwald A, Ott GK, Delabie J, Campo E, Gascoyne RD, Cairo MS, Weisenburger DD, Greiner TC, Gross TG, Chan WC (2012). Molecular distinctions between pediatric and adult mature B-cell non-Hodgkin lymphomas identified through genomic profiling. Blood.

[22] Klapper W, Kreuz M, Kohler CW, Burkhardt B, Szczepanowski M, Salaverria I, Hummel M, Loeffler M, Pellissery S, Woessmann W, Schwanen C, Trumper L, Wessendorf S, Spang R, Hasenclever D, Siebert R, Molecular Mechanisms in Malignant Lymphomas Network Project of the Deutsche K (2012). Patient age at diagnosis is associated with the molecular characteristics of diffuse large B-cell lymphoma. Blood.

[23] Hecht JL, Aster JC (2000). Molecular biology of Burkitt's lymphoma. J Clin Oncol.

[24] Copie-Bergman C, Cuilliere-Dartigues P, Baia M, Briere J, Delarue R, Canioni D, Salles G, Parrens M, Belhadj K, Fabiani B, Recher C, Petrella T, Ketterer N, Peyrade F, Haioun C, Nagel I, Siebert R, Jardin F, Leroy K, Jais JP, Tilly H, Molina TJ, Gaulard P (2015). MYC-IG rearrangements are negative predictors of survival in DLBCL patients treated with immunochemotherapy: a GELA/LYSA study. Blood.

[25] Gagnon MF, Pearce KE, Greipp PT, Xu X, Hoppman NL, Ketterling RP, McPhail ED, King RL, Baughn LB, Peterson JF (2021). MYC break-apart FISH probe set reveals frequent unbalanced patterns of uncertain significance when evaluating aggressive B-cell lymphoma. Blood Cancer J.

[26] McPhail ED, Maurer MJ, Macon WR, Feldman AL, Kurtin PJ, Ketterling RP, Vaidya R, Cerhan JR, Ansell SM, Porrata LF, Nowakowski GS, Witzig TE, Habermann TM (2018). Inferior survival in high-grade B-cell lymphoma with MYC and BCL2 and/or BCL6 rearrangements is not associated with MYC/IG gene rearrangements. Haematologica.

[27] Bertrand P, Bastard C, Maingonnat C, Jardin F, Maisonneuve C, Courel MN, Ruminy P, Picquenot JM, Tilly H (2007). Mapping of MYC breakpoints in 8q24 rearrangements involving non-immunoglobulin partners in B-cell lymphomas. Leukemia.

[28] Otto C, Scholtysik R, Schmitz R, Kreuz M, Becher C, Hummel M, Rosenwald A, Trumper L, Klapper W, Siebert R, Kuppers R, Molecular Mechanisms in Malignant Lymphomas" Network P (2016). Novel IGH and MYC translocation partners in diffuse large B-cell lymphomas genes chromosomes. Cancer.

[29] Oschlies I, Klapper W, Zimmermann M, Krams M, Wacker HH, Burkhardt B, Harder L, Siebert R, Reiter A, Parwaresch R (2006). Diffuse large B-cell lymphoma in pediatric patients belongs predominantly to the germinal-center type B-cell lymphomas: a clinicopathologic analysis of cases included in the German BFM (Berlin-Frankfurt-Munster) Multicenter Trial. Blood.

[30] King RL, Hsi ED, Chan WC, Piris MA, Cook JR, Scott DW, Swerdlow SH (2023). Diagnostic approaches and future directions in Burkitt lymphoma and high-grade B-cell lymphoma. Virchows Arch.

[31] Schmitz R, Wright GW, Huang DW, Johnson CA, Phelan JD, Wang JQ, Roulland S, Kasbekar M, Young RM, Shaffer AL, Hodson DJ, Xiao W, Yu X, Yang Y, Zhao H, Xu W, Liu X, Zhou B, Du W, Chan WC, Jaffe ES, Gascoyne RD, Connors JM, Campo E, Lopez-Guillermo A, Rosenwald A, Ott G, Delabie J, Rimsza LM, Tay Kuang Wei K, Zelenetz AD, Leonard JP, Bartlett NL, Tran B, Shetty J, Zhao Y, Soppet DR, Pittaluga S, Wilson WH, Staudt LM (2018). Genetics and pathogenesis of diffuse large B-cell lymphoma. N Engl J Med.

[32] Khurana A, Mwangi R, Cerhan JR, Cohen JB, Chapman-Fredricks JR, Friedberg JW, Flowers CR, Burack R, Lossos IS, Nastoupil LJ, Feldman AL, Kahl BS, Martin P, Nowakowski GS, Link BK, McDonnell TJ, Inghirami G, Syrbu S, Vij KR, Maurer MJ, Habermann TM, King RL (2023). Comparing clinical characteristics and outcomes of MYC and BCL6 double hit lymphoma (DHL-BCL6) with other aggressive B-cell lymphomas: understanding the impact of new WHO and International Consensus Classifications. Blood.

[33] Li S, Desai P, Lin P, Yin CC, Tang G, Wang XJ, Konoplev SN, Khoury JD, Bueso-Ramos CE, Medeiros LJ (2016). MYC/BCL6 double-hit lymphoma (DHL): a tumour associated with an aggressive clinical course and poor prognosis. Histopathology.

[34] Rosenwald A, Bens S, Advani R, Barrans S, Copie-Bergman C, Elsensohn MH, Natkunam Y, Calaminici M, Sander B, Baia M, Smith A, Painter D, Pham L, Zhao S, Ziepert M, Jordanova ES, Molina TJ, Kersten MJ, Kimby E, Klapper W, Raemaekers J, Schmitz N, Jardin F, Stevens WBC, Hoster E, Hagenbeek A, Gribben JG, Siebert R, Gascoyne RD, Scott DW, Gaulard P, Salles G, Burton C, de Jong D, Sehn LH, Maucort-Boulch D (2019). Prognostic significance of myc rearrangement and translocation partner in diffuse large B-cell lymphoma: a study by the Lunenburg Lymphoma Biomarker Consortium. J Clin Oncol.

[35] Ye Q, Xu-Monette ZY, Tzankov A, Deng L, Wang X, Manyam GC, Visco C, Montes-Moreno S, Zhang L, Dybkaer K, Chiu A, Orazi A, Zu Y, Bhagat G, Richards KL, Hsi ED, Choi WW, van Krieken JH, Huh J, Ponzoni M, Ferreri AJ, Parsons BM, Moller MB, Piris MA, Winter JN, Medeiros LJ, Hu S, Young KH (2016). Prognostic impact of concurrent MYC and BCL6 rearrangements and expression in de novo diffuse large B-cell lymphoma. Oncotarget.

[36] Zhang C, Stelloo E, Barrans S, Cucco F, Jiang D, Tzioni MM, Chen Z, Li Y, Swennenhuis JF, Makker J, Raso-Barnett L, Liu H, El-Daly H, Soilleux E, Shah N, Nagumantry SK, Kyaw M, Prahladan MP, Tooze R, Westhead DR, Feitsma H, Davies AJ, Burton C, Johnson PWM, Du MQ (2024). Non-IG::MYC in diffuse large B-cell lymphoma confers variable genomic configurations and MYC transactivation potential. Leukemia.

[37] Ryan RJ, Drier Y, Whitton H, Cotton MJ, Kaur J, Issner R, Gillespie S, Epstein CB, Nardi V, Sohani AR, Hochberg EP, Bernstein BE (2015). Detection of enhancer-associated rearrangements reveals mechanisms of oncogene dysregulation in B-cell lymphoma. Cancer Discov.

[38] Kunstner A, Witte HM, Riedl J, Bernard V, Stolting S, Merz H, Olschewski V, Peter W, Ketzer J, Busch Y, Trojok P, Bubnoff NV, Busch H, Feller AC, Gebauer N (2022). Mutational landscape of high-grade B-cell lymphoma with MYC-, BCL2 and/or BCL6 rearrangements characterized by whole-exome sequencing. Haematologica.

[39] Wright GW, Huang DW, Phelan JD, Coulibaly ZA, Roulland S, Young RM, Wang JQ, Schmitz R, Morin RD, Tang J, Jiang A, Bagaev A, Plotnikova O, Kotlov N, Johnson CA, Wilson WH, Scott DW, Staudt LM (2020). A probabilistic classification tool for genetic subtypes of diffuse large B cell lymphoma with therapeutic implications. Cancer Cell.

[40] Johnson SM, Umakanthan JM, Yuan J, Fedoriw Y, Bociek RG, Kaiser-Rogers K, Sanmann JN, Montgomery ND (2018). Lymphomas with pseudo-double-hit BCL6-MYC translocations due to t(3;8)(q27;q24) are associated with a germinal center immunophenotype, extranodal involvement, and frequent BCL2 translocations. Hum Pathol.

[41] Rodriguez-Pinilla SM, Dojcinov S, Dotlic S, Gibson SE, Hartmann S, Klimkowska M, Sabattini E, Tousseyn TA, de Jong D, Hsi ED (2024). Aggressive B-cell non-Hodgkin lymphomas: a report of the lymphoma workshop of the 20th meeting of the European Association for Haematopathology. Virchows Arch.

